# Laser-Induced Graphene Electrodes for Flexible pH Sensors

**DOI:** 10.3390/nano14242008

**Published:** 2024-12-14

**Authors:** Giulia Massaglia, Giacomo Spisni, Tommaso Serra, Marzia Quaglio

**Affiliations:** 1 Department of Applied Science and Technology, Politecnico di Torino, Corso Duca Degli Abruzzi 24, 10129 Turin, Italy; giacomo.spisni@polito.it (G.S.); tommaso.serra@polito.it (T.S.); 2 Center for Sustainable Future Technologies, Istituto Italiano di Tecnologia, CSFT@Polito, Via Livorno 60, 10100 Turin, Italy

**Keywords:** bio-electrochemical sensors, flexible sensors, laser-induced graphene material, ultrasonic spray coating, nanostructured active material, pH sensitivity

## Abstract

In the growing field of personalized medicine, non-invasive wearable devices and sensors are valuable diagnostic tools for the real-time monitoring of physiological and biokinetic signals. Among all the possible multiple (bio)-entities, pH is important in defining health-related biological information, since its variations or alterations can be considered the cause or the effect of disease and disfunction within a biological system. In this work, an innovative (bio)-electrochemical flexible pH sensor was proposed by realizing three electrodes (working, reference, and counter) directly on a polyimide (Kapton) sheet through the implementation of CO_2_ laser writing, which locally converts the polymeric sheet into a laser-induced graphene material (LIG electrodes), preserving inherent mechanical flexibility of Kapton. A uniform distribution of nanostructured PEDOT:PSS was deposited via ultrasonic spray coating onto an LIG working electrode as the active material for pH sensing. With a pH-sensitive PEDOT coating, this flexible sensor showed good sensitivity defined through a linear Nernstian slope of (75.6 ± 9.1) mV/pH, across a pH range from 1 to 7. We demonstrated the capability to use this flexible pH sensor during dynamic experiments, and thus concluded that this device was suitable to guarantee an immediate response and good repeatability by measuring the same OCP values in correspondence with the same pH applied.

## 1. Introduction

Continuous remote clinical monitoring and real-time diagnostics represent pivotal elements for tomorrow’s personalized medicine. Non-invasive wearable devices and sensors are valuable diagnostic tools for real-time monitoring of physiological and bio-kinetic signals, without interrupting or limiting the user’s movement [1,2,3,4,5,6,7,8,9,10]. Real-time information reflecting the physiological and health status of the individual is collected by continuous monitoring of certain biomarkers in biological fluids, such as sweat, tears, and saliva [11,12,13,14,15,16,17]. All these efforts turn out to be pivotal for personalized medicine, providing the operators with continuous access to information about the individual’s health, thus preventing interventions in complications that may arise [2,18,19,20]. To this purpose, several works in the literature focused their attention on the development of wearable (chemical) biosensors that are able to detect multiple entities, such as pH, Na^+^, K^+^, Ca^2+^, NH_4_^+^, Cl^−^, glucose, dopamine, urea, etc. [1,5,21,22,23,24,25,26,27,28,29,30,31].

Among them, the detection of pH has attracted the interest of many researchers due to its importance in defining health-related biological information. Alterations and variations in pH can be considered the cause or the effect of disease and disfunction within a biological system. Skin lesions, such as irritant contact dermatitis, acne vulgaris, and atopic dermatitis, induce abnormal sweat pH.

Moreover, many cellular processes and enzymatic reactions are directly dependent on pH. Different studies demonstrated how acidic environments lead to inflamed blood cells, reducing the oxygen levels, altering the cells’ metabolic activity, and hindering the function of DNA and respiratory enzymes, which can be responsible for kidney, liver, and sweat gland failure [32,33,34]. One study investigated the direct correlation between a decrease in pH from 6.9 to 6.5 and the detriment of living cells and the consequent tumor onset [35].

It becomes apparent that there is a direct correlation between the pH levels of body fluids and health, rendering the development of wearable sensors capable of detecting pH absolutely necessary, thus also enabling the early detection of many diseases.

Therefore, more effort must be made to select proper sensitive materials for pH detection and to design congruently the electrode that ensures the proper flexibility required for a wearable sensor.

The most common pH sensor is represented by a glass electrode that exhibits some inherent disadvantages, like high-temperature instability, acid-base error, high impedance, and the complexity in miniaturizing the electrode itself to guarantee its fit [36,37].

Alternatively, with traditional glass electrodes, current research on pH-sensing technologies is oriented towards optical, transistor-based, or electrochemical systems. Depending on the technology, these novel approaches offer advantages in terms of sensitivity and selectivity towards specific ions and potential for miniaturization and real-time monitoring [38,39,40,41,42].

Optical pH sensing is based on optical properties variation in pH-sensitive materials [38,40]. The sensing material, which might consist of dyes or inorganic composites, when in contact with the solution to be analyzed (the analyte), experiences a measurable shift in its optical properties (such as color, absorbance, or fluorescence). These sensors offer good sensitivity and precision, but usually feature a narrow operative range and require an optical readout setup.

Instead, transistor-based pH sensors normally feature a H+-sensitive gate electrode, which transduces the pH value into the conductivity of the transistor dielectric [43,44]. When the gate electrode interacts with the analyte, the variation in the transistor dielectric conductivity can be measured by applying a voltage between the source and drain electrodes. Despite the earlier stage of development and the need to overcome stability and fabrication complexity challenges, this technology represents an interesting solution for several pH-sensing applications [38,43].

On the other hand, electrochemical pH sensors rely on potentiometric or voltametric measurements performed on a pH-sensitive electrode. Potentiometric electrochemical sensors measure the potential arising between two electrodes (the working, WE, and reference, RE) when immersed in the analyte. Instead, voltametric sensors require a three-electrode configuration (a counter electrode, CE, is added), through which a current is measured as a result of an imposed potential. This electrochemical pH-sensing technology is of particular interest for biomedical applications thanks to the compact and integrable design and the relatively low cost.

In several studies [45,46,47,48], electrochemical pH sensors find applications in point-of-care diagnostics and in wearable monitoring devices. For such applications, pH-sensing devices must be flexible [41,49,50] and satisfy stability and biocompatibility requirements. These aspects can be finely tuned by selecting the electrodes’ materials.

In addition, active sensitive materials for the detection of pH are the other important parameter to make the final sensors suitable for the detection of pH. An important option for wearable pH sensors is achieved by intrinsic conductive materials, such as Polyaniline (PANI) [49] or Poly(3,4-ethylenedioxythiophene) PEDOT:PSS, due to their ion-exchanging features [41].

Different works in the literature [49] demonstrate how PANI membranes are one of the most widely used pH-sensitive materials, due to their strong pH sensitivity [51,52], good ionic and electronic conductivity [53], chemical stability, and low cost. Furthermore, PANI is involved in the design of wearable pH sensors also due to its easy deposition and larger mechanical flexibility. The main constraint of PANI is represented by the biotoxicity induced by possible impurities including benzidine, which affects the confirmed biocompatibility of PANI [54] and hinders its application as a sensitive material for bio electrochemical devices.

To overcome these limitations of PANI, active PEDOT:PSS-based materials are valid alternatives to develop sensors for pH detection, ensuring a relatively good stability and adjustable conductivity [55,56].

PEDOT:PSS also provides biocompatibility, high mechanical flexibility, and the possibility of realizing composites and nanostructures that further enhance its outstanding pH sensitivity [57,58,59].

With a view to realizing inexpensive and disposable pH-sensing devices, easily scalable and low-cost fabrication processes are preferred. For this reason, a deposition of commercial PEDOT:PSS represents a simpler yet effective solution regarding electrochemical polymerization or other composite preparations.

To provide electrical conductivity and mechanical support, PEDOT:PSS can be deposited on carbon-based electrodes obtained from laser-induced graphitization (LIG) of flexible polymer material [60,61,62,63,64,65,66,67,68,69,70,71,72,73,74]. LIG electrodes represent an ideal solution for the realization of (bio) electrochemical sensors, as they provide good electrical conductivity, mechanical flexibility, chemical stability, and biocompatibility. At the same time, the large surface area and the fabrication process tuneability can be exploited to tailor the sensor’s detection performance and characteristics to the desired field of application [60,61].

In this work, a new design for flexible wearable (bio) electrochemical sensors is proposed by realizing the three electrodes (working, reference, and counter) directly on a polyimide (Kapton) sheet through the implementation of CO_2_ laser writing, which locally converts the polymeric sheet into a laser-induced graphene material (LIG electrodes).

In this way, it was possible to optimize the geometrical and spatial distribution of all three electrodes, mimicking the commercial screen-printed electrodes (SPEs, MetroOhm Dropsens 110D, Oviedo, Asturias (Spain)). This approach allowed the realization of LIG electrodes with a coplanar distribution directly on a Kapton sheet, thus preserving the high mechanical flexibility inherent in the polymeric sheet [63]. Moreover, a uniform distribution of nanostructured PEDOT:PSS was deposited via ultrasonic spray coating (USC) onto the LIG working electrode as an active material for pH sensing, leading to the final flexible sensor named PEDOT-LIG. The present work, first of all, confirmed the electrochemical response achieved when PEDOT:PSS was provided as a sensitive material for pH sensors, whose model is thoroughly explained by our previous work [75]. Moreover, it is important to emphasize how this electrochemical response was also found to be valid when LIG electrodes are used for pH sensing. Open Circuit Voltage methods were implemented to evaluate the potentiometric response of the PEDOT-LIG sensor compared with the one reached when nanostructured PEDOT:PSS was deposited by a USC process onto commercial Dropsens electrodes. It was possible to demonstrate the good sensitivity of the PEDOT-LIG sensor, which ensures a Nernstian slope of (75.6 ± 9.1) mV/pH. Such good results open the door to the possibility of applying PEDOT-LIG sensors as effective pH sensors. In this work, this was simulated through the implementation of dynamic experiments during which the pH was continuously modified, allowing us to define the real-time response of flexible PEDOT-LIG as a pH sensor.

## 2. Materials and Methods

### 2.1. Design and Realization of LIG Electrodes

LIG electrodes were fabricated using a commercial laser scriber by CO_2_ Laser Veronesi equipped with a pulse CO_2_ source (Maximum nominal Power P = 10 W, and wavelength τ close to 10.6 µm). The design of LIG electrodes was achieved by implementing CO_2_ laser writing under ambient conditions and with the main purpose of mimicing the same configuration and spatial distribution of electrodes and the same dimensions and areas, followed by commercial screen-printed carbon electrodes (Dropsens electrodes, purchased by Metrohm, 110D).

The employed laser parameters for electrode fabrication are reported in Table 1.

Commercial Dropsens electrode 110D was constituted by 3 coplanar electrodes: (i) working electrode made of carbon-based material; (ii) reference electrode, composed of silver/silver chloride (Ag/AgCl); and (iii) counter electrode, consisting of carbon-based electrodes.

The final LIG electrode is composed of 3 coplanar LIG electrodes: (i) working electrode, whose diameter is 0.4 cm, (ii) reference electrode, and (iii) counter electrode. The main difference between LIG electrodes and the Dropsens electrode can be identified in the reference electrode that is made of LIG and silver chloride (Ag/AgCl) in the commercial electrode, as well as the excellent mechanical properties in terms of the flexibility provided by the new electrode configuration.

### 2.2. Nanostructured Deposition of PEDOT:PSS Obtained by Ultrasonic Spray Coating Process

An ultrasonic spray coating (Nadetech Ultrasonic Spray Coater, purchased from Nadetech Innovations, Navarra, Spain) process was employed to directly deposit the nanostructured layer of PEDOT:PSS on both of electrode’s configurations, LIG electrodes, and DropSens electrodes. This process ensured a uniform deposition of the active sensing material on the working electrode, without requiring the utilization of a binder to guarantee the proper adhesion of PEDOT:PSS onto working electrode. As deeply investigated by Spisni et al. [76], a water-based spraying solution of 1 mg/mL of PEDOT and 1.6 mg/mL of PSS was prepared to finely tune the amount of PEDOT deposited, simultaneously guaranteeing spatial uniformity. A target PEDOT:PSS deposited amount of 200 µg/cm^2^ was obtained by controlling the deposition parameters in the ultrasonic spray coating equipment. The deposition was performed by operating at a nozzle-to-deposition plate distance of 70 mm, a 10 mL/h flow rate, and a nozzle speed of 400 mm/min. These parameters allowed us to determine the deposited material amount by estimating the material flux (flow rate and nozzle speed) and the deposition duration (nozzle speed and number of passes). During the deposition, an aluminum foil mask was also employed to limit the PEDOT:PSS deposition to the working electrode area.

Moreover, the ultrasonic spray coating process allowed us to crosslink the deposited material on-site by setting the temperature of the deposition plate. The crosslinking step was required to avoid the dissolution of the nanostructured PEDOT:PSS layer once put in contact with water-based solutions [77].

A plate temperature of 120 °C was set to simultaneously ensure water evaporation and material crosslinking. The final crosslinked nanostructured layer, containing an amount of PEDOT close to 200 μg/cm^2^, was employed as an active material for pH sensing.

### 2.3. Design of LIG-PEDOT pH Sensors

**Figure 1:**
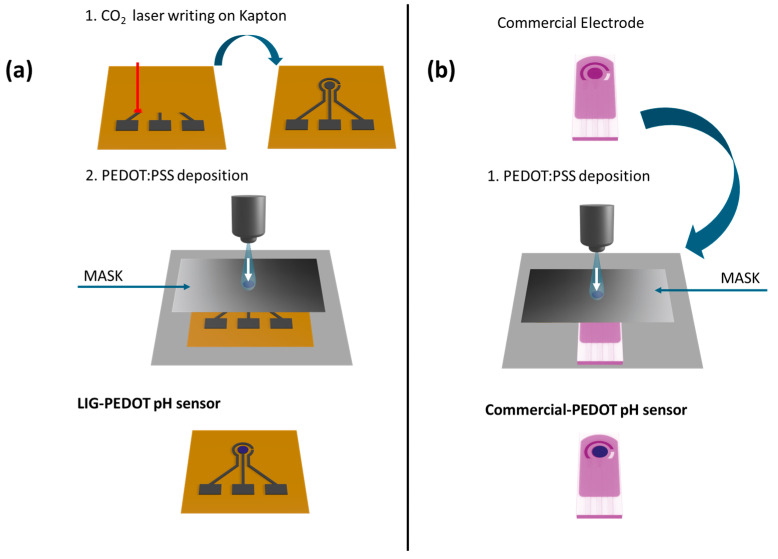
The schematic representation of the process workflows proposed: in (**a**), the workflow referring to the realization of the LIG-PEDOT pH sensor is sketched, while in (**b**), the one followed for fabricating the commercial-PEDOT pH sensor is represented.

The described process allowed the deposition of a PEDOT nanostructured layer on both the LIG working electrode, realizing the design of a flexible PEDOT-LIG pH sensor, and on the Dropsens working electrode (commercial-PEDOT pH sensor, purchased from MetroOhm Dropsens, Oviedo, Asturias (Spain)). Figure 1a reports the process flow implemented to design LIG-PEDOT pH sensors, and Figure 1b describes the one to fabricate commercial-PEDOT pH sensors.

During the whole experiment, the performance of the LIG-PEDOT pH sensors was compared with that reached with the commercial-PEDOT pH sensors.

### 2.4. Moprhological and Electrochemical Characterization

To evaluate the morphological properties of LIG electrodes and surface morphology of the nanostructured PEDOT layer, a Field Emission Scanning Electron Microscope (FESEM, ZEISS Supra 40, Carl Zeiss AG, Oberkochen, Germany) was employed. Moreover, Raman spectroscopy (inVia™ Qontor, Renishaw, Wotton-under-Edge, UK) was implemented with the main aim of demonstrating the effective role of the USC process in the formation of the quinoid structure of the PEDOT chain. Typically, the PEDOT chain exists in both benzoid and quinoid structures simultaneously [78,79,80].

The benzoid structure is known as a coil-like conformation, characterized by a deviation among each consecutive thiophene plane. Moreover, in this chain configuration, two adjacent thiophene planes are connected by C-C single bonds, characterized by a lower density of conjugated π-electrons [79]. In contrast, the quinoid structure features an elongated conformation, where the orientation of two adjacent thiophane rings remain almost in the same plane. Also, the presence of C=C double bonds along the polymer backbone determines a delocalization of conjugated π-electrons along the PEDOT chain.

Therefore, the prevalence of the quinoid structure compared to the benzoid one provides the efficient electrical conductivity of the polymeric layer. These physical properties, combined with the high porosity inherent in the final PEDOT-LIG pH sensors, may improve the overall performance in terms of sensitivity and linear response compared to the commercial electrode.

Electrochemical Impedance Spectroscopy (EIS) was performed to confirm the expected electrochemical response of PEDOT-LIG pH sensors, according to which the higher the pH, the higher the value of capacitance at the interface (Cdl) between the PEDOT layer surface and the electrolyte. EIS characterizations with a frequency range from 100 kHz to 200 mHz and a perturbation signal amplitude of 10 mV were implemented, and 3 values of pH, equal to 4, 7, and 10, were tested.

Furthermore, Open Circuit Voltage (OCP) measurements were acquired with a Palmsens Potentiostat (PalmSens BV, Utrecht, The Netherlands) to evaluate the potentiometric response of PEDOT-LIG, using water-based electrolytes at different pH. All obtained results were compared with the ones acquired with commercial-PEDOT pH sensors.

Different pH values of solutions were defined with a pH meter (Hanna, HI5221, Padova, Italy) and were adjusted adding either HCl or NaOH to deionized water, thus leading to different pH values in the range from 7 to 1, representative of a neutral environment and a very acidic one.

The same characterization was conducted both in static and dynamic modes. In the static mode, a new electrolyte drop was deposited on the two electrodes (PEDOT-LIG and commercial-PEDOT pH sensors) for each pH value. However, in the dynamic mode, the pH was varied in the same solution in which the electrodes were immersed. Dynamic characterizations allowed us to demonstrate the capability of LIG-PEDOT pH sensors to detect all pH variations in real time.

## 3. Results and Discussion

### 3.1. Morphological Properties of Flexible pH Sensors

**Figure 2:**
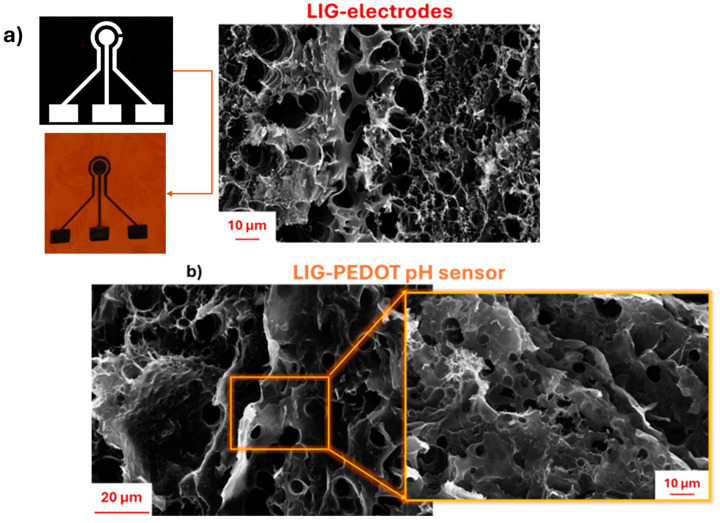
(**a**) Morphological properties of LIG electrodes realized on Kapton sheet by implementing CO_2_ laser writing; (**b**) morphological features of 200 μg/cm^2^ of PEDOT onto LIG electrode.

Figure 2a reports the morphological properties of LIG electrodes directly realized onto a Kapton sheet, by implementing the CO_2_ laser writing process, able to ensure local transformation of polymers into LIG material following a defined design, as highlighted by CAD images.

Moreover, Figure 2a allowed us to confirm the typical structure of LIG materials, which are characterized by a uniform porosity structure that resembles a honeycomb, thus reflecting the conventional shape of graphene. From the FESEM images in Figure 1, it was possible to observe that pore diameters fell in the µm to tens-of-µm range. These dimensions are fully compatible with the diffusion of H^+^ ions (in the form of hydronium ions), with the latter being more than four orders of magnitude smaller than the observed pore sizes. The observed pore structure is also coherent with morphologies reported in other literature works employing LIG-based electrodes for sensing applications [60,61,64,65,66,67,68,69,70].

From the FESEM images, it was possible to also observe continuous interconnections among all pores distributed along the surface of PEDOT-LIG pH sensors, suitable for enhancing the interaction between analyte and surface, preserving the mechanical properties in terms of flexibility of final pH sensors. In contrast, the morphological features of the carbon-based commercial working electrode (Dropsens electrode) showed a uniform and continuous distribution of carbon-based material. The features may be detrimental for the diffusion of H+ ions, combined with worse mechanical properties of the entire commercial Dropsens, which result in it being fragile.

This feature plays a pivotal role in enhancing the ion and electron transport through this pore area. The same honeycomb-like structure of the porosity was preserved even after the disposition of the nanostructured PEDOT:PSS layer, realized by the USC process (Figure 2b).

Furthermore, it was possible to appreciate a uniform deposition of PEDOT on LIG electrodes, preserving the interconnections among pores, thus favoring the mass electrolyte transfer into the active materials during electrochemical reactions, avoiding as much as possible the swelling and cracking of nanomaterials and simultaneously improving ionic conductivity. This latter consideration can positively affect the final pH sensitivity of LIG-PEDOT pH sensors.

Raman spectroscopy was implemented to assess the arrangement of PEDOT chains inside the nanostructured layer of PEDOT:PSS, deposited via the USC process, onto LIG electrodes. This allowed us to verify whether the quinoid conformation of the chains prevailed over the benzoid conformation.

**Figure 3:**
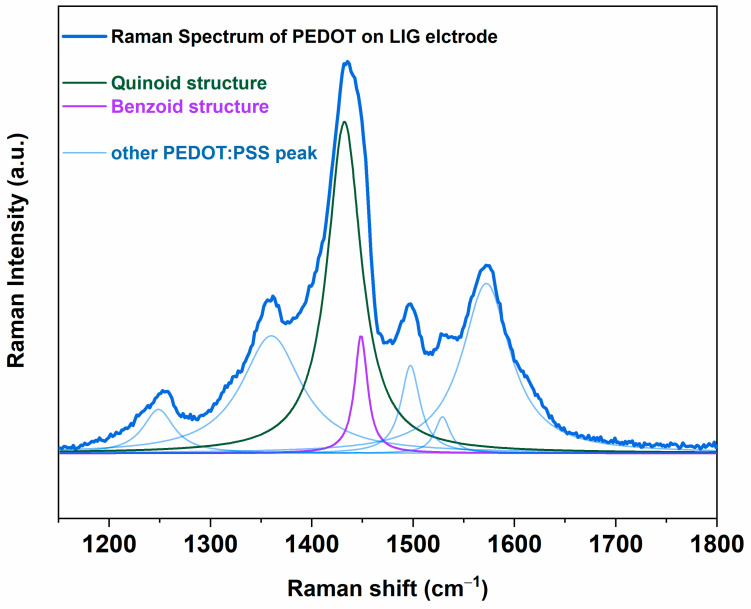
Raman spectrum of PEDOT:PSS nanostructured layer deposited onto LIG electrodes by implementing USC process. It is possible to underline the prevalence of the benzoid group (purple line) over the quinoid one (green line).

In particular, Figure 3 presents the Raman spectrum of PEDOT:PSS directly deposited onto LIG electrodes by the USC process. It was important to underline that typically the PEDOT chain exists in both benzoid and quinoid structures simultaneously and the typical absorption peak at 1450 cm^−1^ was assigned to stretching vibration of C=C [78].

As the Raman peak characteristic of C=C shifted towards lower wavenumbers, it was possible to confirm the prevalence of the quinoid PEDOT conformation over the benzoid form.

By analyzing the Raman spectrum reported in Figure 3, it was possible to appreciate that the absorption peak, due to the stretching vibration of C=C, was found at a Raman shift of (1432.2 ± 0.5) cm^−1^, which is lower than the typical value for the C=C bond close to 1450 cm^−1^.

It is also interesting to consider the ratio among the areas associated with the two conformational fingerptint peaks (A_quinoid_/A_benzoid_), which provides a quantification of the relative presence of the two structural conformations [78,79].

Literature works report values of A_quinoid_/A_benzoid_ ∼0.2 for pristine PEDOT:PSS, which may rise to A_quinoid_/A_benzoid_ ∼2.6 after solvent treatment.

In the case of the spectrum presented in Figure 3, a A_quinoid_/A_benzoid_ ∼7.4 ratio was observed. This latter result allowed us to confirm the prevalence of the quinoid over benzoid form when the nanostructured layer of PEDOT was deposited onto LIG electrodes by the USC process, without the necessity to implement any further solvent treatment.

The combination of the morphological features of the final LIG-PEDOT pH sensors, such as high surface area and great intrinsic porosity, thus assuming a honeycomb-like conformation, and the improved electrical conductivity of the nanostructured PEDOT layer, due to the presence of quinoid conformation of the PEDOT chain, can result in it being effective in the enhancement of the LIG-PEDOT pH sensor.

In particular, the proposed final pH sensor is characterized by better mass transport of the electrolyte and ions from the electrolyte to the electrode surface due to the high interconnected porosity and better electrical conductivtiy due to a linear arrangement of PEDOT (quinoid conformation) chains.

All of this could result in better sensitivity of the sensor itself in detecting pH changes.

### 3.2. Electrochemical Characterization of All pH Sensors

**Figure 4:**
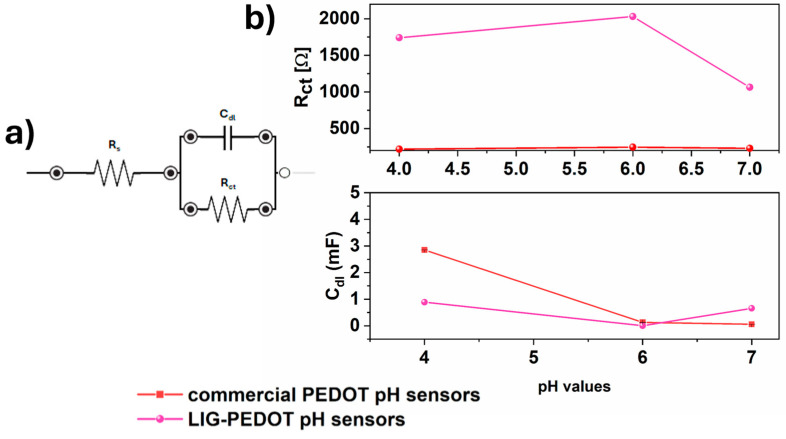
(**a**) Equivalent circuit used to determine electrochemical parameters; (**b**) double-layer capacitance and charge transfer resistance variation of the electrochemical sensor with pH values.

EIS characterizations were performed to confirm the electrochemical response of flexible pH sensors, as widely delineated by Verpoorten et al. [75]. Figure 4 reports the trends of charge resistance (R_ct_) and double-layer capacitance (C_dl_) as a function of different pH values. R_ct_ and C_dl_ values were defined by extrapolating an equivalent electrical circuit, as depicted in Figure 4a.

R_ct_ is a charge transfer resistance describing the resistance faced by the electrons moving from the electrolyte solution to the electrodes’ surface. In contrast, C_dl_ represents the double-layer capacitance that is achieved at the interface between the electrodes and the electrolyte solution. It was possible to appreciate that all obtained results confirmed two linear behaviors for both the capacitance of interfaces and charge transfer resistance, as delineated by our previous work [75].

As the pH values increased, it was possible to observe a decrease in C_dl_ and an increase in Rct, thus confirming the model provided in [75] to explain the electrochemical mechanisms behind the response of pH sensors.

Moreover, it can be seen that both R_ct_ and C_dl_ values of the LIG-PEDOT pH sensors showed greater variation among pH values with respect to the commercial PEDOT sensors, as reported in Figure 4b.

Therefore, it can be stated that the morphological characteristics of the LIG-PEDOT sensor ensure better sensitivity due to the porous structure, which improves the ion and electron transportation through its pore area.

At the same time, the morphological features of LIG-PEDOT pH sensors guarantees a huge presence of -SO^3−^ and -OH groups, which act as proton acceptors on the surface of the electrode itself, thus leading to the minimum value of C_dl_ and blocking all the protons near the surface when the pH is equal to 6.

Open Circuit potential measurement (OCP) was implemented to evaluate all proposed sensors as effective potentiometric pH sensors. In particular, the variations in OCP values were correlated with the pH changes from 1 to 7, mimicking strongly acidic environments, which are found to be the most representative values of different body fluids.

A linear fitting was implemented to define the linear Nernstian response for LIG-PEDOT and PEDOT commercial pH sensors for what concerns the response evaluation of both potentiometric sensors. The Nernstian response was achieved over the pH range from 1 to 7 with a 100 μL drop of electrolyte solution covering all planar electrodes.

**Figure 5:**
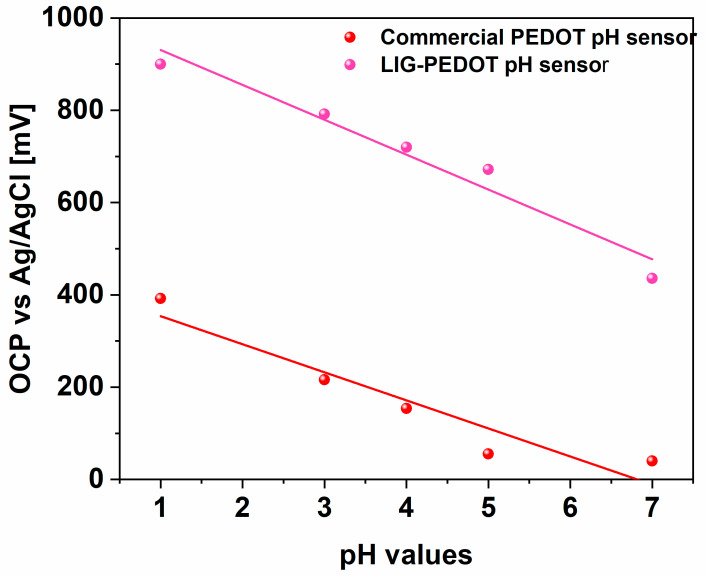
OCP measurements conducted at different pH values, defined in the range from 1 to 7, mimicking the acidic environment. Experimental data for LIG-PEDOT pH sensors (pink dot) were compared with those for commercial-PEDOT (red dot), highlighting a linear pH response (pink line for LIG-PEDOT pH sensor and red line for commercial PEDOT, respectively).

By analyzing Figure 5, it was possible to appreciate the linear Nernstian response of LIG-PEDOT pH sensors with a pH sensitivity close to (−75.6 ± 9.1) mV/pH, slightly better than the one achieved with commercial PEDOT pH sensors ((−60.8 ± 11.3) mV/pH).

These pH sensitivity values can be compared with other pH-sensing devices presented in the literature [47,64,65,66,67,68,69,70,71,72,73,74], which are summarized in Table 2.

It can be observed how the LIG-PEDOT pH sensors presented in this work feature a sensitivity compatible with the LIG/PEDOT:PSS/PANI devices realized by [74], or slightly higher than that proposed by [68], where Riboflavin acts as pH-sensitive material in place of PEDOT:PSS. The sensitivity performances can also be compared to other works employing combinations of PANI, multi-walled carbon nanotubes (MWCNTs), or iridium oxide (IrOx).

The LIG-PEDOT pH sensors presented in this work provided the advantage of employing PEDOT:PSS as the sole pH-sensitive material, reducing the complexity of the fabrication process. Also, with respect to commercial devices based on flat electrodes, the porous structure of the LIG electrodes increases the surface area exposed to the analyte. This results in an enhanced sensitivity, observable by comparing the response from the commercial PEDOT and the LIG-PEDOT pH sensors.

These latter results, which are in line with the electrochemical responses described previously, reinforced the importance of the morphological properties offered by the LIG-PEDOT pH sensor.

The high surface area of the LIG-PEDOT sensor, indeed, ensures greater exposure of the SO^3−^ anionic groups, available for the recognition of H^+^ present in the electrolyte solution, thus converting the ionic signal into a stronger electronic signal.

Furthermore, the presence of the quinoid PEDOT structure, characterizing the nanostructured layer onto the LIG electrode, improved the electrical conductivity, thus leading to a larger number of conducting pathways for electron and ion transport.

Due to the high pH sensitivity of new flexible LIG-PEDOT pH sensors, a dynamic test was implemented to demonstrate their capability to detect all pH variations.

LIG-PEDOT pH sensors were immersed in a water-based electrolyte, and pH values were continuously varied by adding different amounts of NaOH or HCl components to simulate acidic and basic environments.

**Figure 6:**
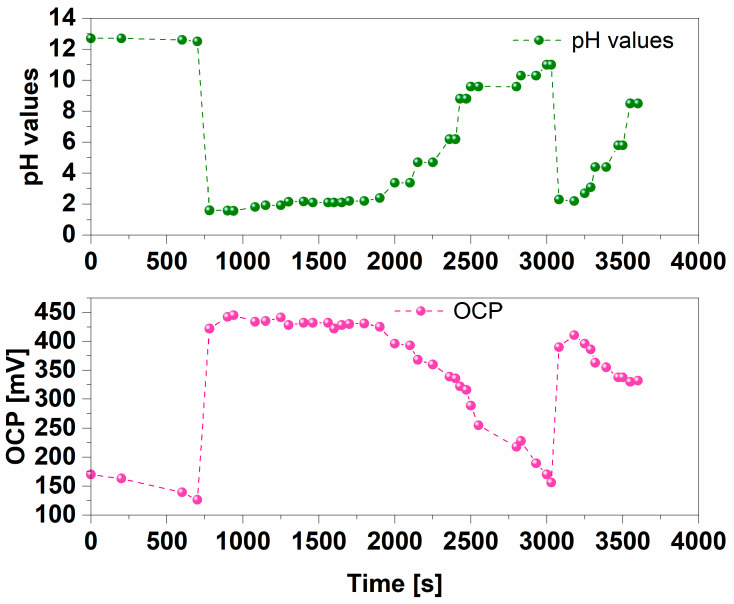
OCP measurements conducted at different pH values in a dynamic way. LIG-PEDOT pH sensors were immersed in the electrolyte solution, and pH values were continuously modified by adding NaOH and HCl to move from a basic environment to a strong acidic one.

Figure 6 reports on the capability of LIG-PEDOT pH sensors to ensure a real-time response to the pH changes, in terms of OCP variations.

Furthermore, it was possible to confirm the linear response observed when the pH changed from an acidic environment (pH = 1) to a neutral pH (pH = 7), similarly to what was achieved during the previous static experiments.

All obtained results allowed us to demonstrate the capability of LIG-PEDOT pH sensors to monitor pH changes in real time in the form of OCP variations. The reversibility of such OCP changes was also confirmed, i.e., a certain pH value had a unique correspondence to a certain OCP value. To appreciate this behavior of the nanostructured PEDOT:PSS layer, the two regions corresponding to pH increase from 2 to 8 are to be analyzed. These two pH reductions were made in two distinct time intervals, (600 ÷ 2400) s and (3100 ÷ 3500) s, and were separated by a period of time during which the sensor was kept at high pH values. In the two regions, the OCV increases with the same trend, showing high reproducibility and stability of the OCV versus pH values. These results demonstrate the stability of the nanostructured PEDOT:PSS layer over time.

Finally, it is very important to point out the opposite trend for OCP variations achieved when pH values were changed. When a strongly alkaline environment was provided to the LIG-PEDOT pH sensor, with pH values close to 12, it was possible to identify the minimum OCP value (≈150 mV) achieved.

A drastic drop in pH values, corresponding to the exposure to a strongly acidic environment (pH equal to 2), induced a sudden increase in measured OCP (reaching a value close to 450 mV).

This behavior can be explained by considering the model widely discussed in our previous work [48] and defining the pH = 1 to pH = 7 range as the condition for which C_dl_ increases as the -OH groups of the PEDOT begin to release protons to compensate for the presence of OH^−^ in the solution.

In contrast, as the environment moves towards strongly alkaline environments (pH ≥ 10), that is, when the number of OH^-^ ions in solutions increases, the -OH groups of PEDOT are unable to compensate for the ions in solutions, which induces a decrease in measured OCP values.

By analyzing the real-time response of LIG-PEDOT pH sensors, it was possible to demonstrate how the higher the pH values are, the lower the achieved OCP value is.

All the results obtained with the flexible LIG-PEDOT as a pH sensor demonstrated the effectiveness of the sensor itself.

The device guaranteed an immediate response, linking the measured OCP with the values of pH of the electrolyte where sensor was immersed.

It was possible to verify that the minimum measured OCP values corresponded to the maximum pH applied and vice versa.

Finally, the repeatability of the LIG-PEDOT pH sensor was demonstrated by measuring the same OCP values in correspondence with the same pH applied. These observations highlighted how the LIG-PEDOT pH sensor was able to provide a reliable readout of the analyte pH during a continuous operation of one hour.

## 4. Conclusions

The development of new flexible pH sensors was proposed by realizing three LIG- working, reference, and counter electrodes, which were spatially co-planar distributed. To this purpose, the CO_2_ laser writing process was implemented to locally convert a Kapton sheet into LIG materials, thus preserving its intrinsic mechanical flexibility. Moreover, to ensure the effectiveness of these flexible pH sensors, a nanostructured layer was directly deposited onto the LIG working electrode, obtaining the final flexible LIG-PEDOT pH sensor. It was possible to demonstrate a high pH sensitivity ensured by these devices. Indeed, the LIG-PEDOT pH sensor ensured a linear Nernstian response with a pH sensitivity close to (−75.6 ± 9.1) mV/pH, which was slightly better than the one achieved with the commercial PEDOT pH sensors ((−60.8 ± 11.3) mV/pH). All obtained results allowed us to define the effectiveness of this flexible pH sensor.

Indeed, the device guaranteed an immediate response, linking the measured OCP with the values of pH of the electrolyte where the sensor was immersed.

It was possible to verify that the minimum measured OCP values correspond to the maximum pH applied and vice versa.

Finally, the repeatability of the LIG-PEDOT pH sensor was demonstrated by measuring the same OCP values in correspondence with the same pH applied.

## Figures and Tables

**Table 1:** Laser parameters employed in the fabrication of LIG electrodes.

Laser Parameter	Value
Power	70% (equal to around 7 W)
Pulse frequency	4 kHz
Scan velocity along the *x*-axis	125 mm/s
Line spacing along the *y*-axis	20 µm

**Table 2:** Comparison with electrochemical pH sensors based on LIG or PEDOT:PSS from the literature.

Sensor	pH Sensitivity (∆mV/pH)	Working Range (pH)	Ref.
Au/PEDOT:PSS/polyaniline emeraldine	47.33	4–10	[47]
LIG/Riboflavin	56	2.8–8	[64]
PEDOT:PSS/IrOx	81 ± 2	3–11	[71]
PANI/PEDOT:PSS	56 ± 7	3–7	[72]
Cotton/PEDOT-MWCNT/PANI	61 ± 2	2–12	[73]
LIG/PEDOT:PSS/PANI	75.06	4–7	[74]
DropSens 110D/PEDOT:PSS	60.8 ± 11.3	1–7	This work
LIG/PEDOT:PSS	75.6 ± 9.1	1–7	This work

## Data Availability

Data are contained within the article.
